# The Role of Sodium-Dependent Glucose Transporter 1 and Glucose Transporter 2 in the Absorption of Cyanidin-3-*O*-β-Glucoside in Caco-2 Cells

**DOI:** 10.3390/nu6104165

**Published:** 2014-10-13

**Authors:** Tang-Bin Zou, Dan Feng, Gang Song, Hua-Wen Li, Huan-Wen Tang, Wen-Hua Ling

**Affiliations:** 1 School of Public Health, Guangdong Medical College, Dongguan 523808, China; E-Mails: zoutb@163.com (T.-B.Z.); 1572@gdmc.edu.cn (G.S.); chineseli@163.com (H.-W.L.); gdmcthw@126.com (H.-W.T.); 2 Guangdong Provincial Key Laboratory of Food, Nutrition and Health, Department of Nutrition, School of Public Health, Sun Yat-Sen University, Guangzhou 510080, China; E-Mail: crystal031015@163.com

**Keywords:** Cy-3-G, absorption, siRNA, SGLT1, GLUT2

## Abstract

Anthocyanins have multiple biological activities of benefit to human health. While a few studies have been conducted to evaluate the bioavailability of anthocyanins, the mechanisms of their absorption mechanism remain ill-defined. In the present study, we investigated the absorption mechanism of cyanidin-3-*O*-β-glucoside (Cy-3-G) in human intestinal epithelial (Caco-2) cells. Cy-3-G transport was assessed by measuring the absorptive and efflux direction. Inhibition studies were conducted using the pharmacological agents, phloridzin, an inhibitor of sodium-dependent glucose transporter 1 (SGLT1), or phloretin, an inhibitor of glucose transporter 2 (GLUT2). The results showed that phloridzin and phloretin significantly inhibited the absorption of Cy-3-G. In addition, Caco-2 cells transfected with small interfering RNA (siRNA) specific for SGLT1 or GLUT2 showed significantly decreased Cy-3-G absorption. These siRNA transfected cells also showed a significantly decreased rate of transport of Cy-3-G compared with the control group. These findings suggest that Cy-3-G absorption is dependent on the activities of SGLT1 and GLUT2 in the small intestine and that SGLT1 and GLUT2 could be a limiting step for the bioavailability of Cy-3-G.

## 1. Introduction

Anthocyanins, a class of polyphenols, are present in the pigments of numerous colorful fruits and vegetables [1]. Interest in using anthocyanins as valuable natural alternatives to synthetic food colorings has increased in recent years. Increasing evidence shows that anthocyanins have potential free radical-scavenging activities, preventing low-density lipoprotein oxidation and giving rise to beneficial effects on cardiovascular diseases, obesity and inflammation [2,3,4,5]. Our group has demonstrated that anthocyanins play an important role in attenuating the severity and reducing the risk of atherosclerosis [6,7,8], consistent with the observations made by other groups [9].

It is well known that the effectiveness of anthocyanins *in vivo* mainly depends on their absorption, metabolism and excretion properties [10]. Although the bioavailability properties of anthocyanins have been studied [11,12,13], the mechanisms of their bioavailability remain to be identified. While the long-term belief has been that anthocyanins could only be absorbed in their aglycone forms, derived from studies with quercetin [14], which is of a similar basic structure, recent attention has been turned to the absorption of anthocyanins. Several studies have shown that anthocyanins are rapidly absorbed in the stomach and small intestine [15]. Intact anthocyanins were also detected in the plasma and urine of human subjects and rats [16,17]. Although the absorption mechanism of anthocyanins in the stomach is unclear, Passamonti *et al.* [18] suggested the involvement of an anion translocator, such as bilitranslocase, expressed in the gastric epithelium. It is not clear whether sodium-dependent glucose transporter 1 (SGLT1) and glucose transporter 2 (GLUT2) are involved in the intestinal absorption of anthocyanins.

Cyanidin-3-*O*-β-glucoside (Cy-3-G), the most representative dietary anthocyanin, exists widely in mulberry and other higher plants [19,20,21]. In this study, we examined the absorption mechanism of Cy-3-G in the small intestine, with respect to the role of the glucose carrier SGLT1 and GLUT2 in the transportation of cyanidin glycosides across the intestinal brush border membrane using a Caco-2 cell model. A better understanding of the mechanisms of anthocyanin absorption would be helpful in optimizing the application of nutrients in oxidative-induced diseases.

## 2. Experimental Section

### 2.1. Materials

Cy-3-G (purity > 97%) was kindly provided by Polyphenol AS (Sandnes, Norway). Caco-2 cells were obtained from the American Type Culture Collection (ATCC, Rockville, MD, USA). Dulbecco’s modified eagle’s medium (DMEM, high-glucose), nonessential amino acids, phloridzin and phloretin were purchased from Sigma-Aldrich (St. Louis, MO, USA). Penicillin-streptomycin, fetal bovine serum, 0.25% trypsin-EDTA solution and 3-(4,5-dimethylthiazol-yl)-diphenyl tetrazolium bromide (MTT) were obtained from Gibco BRL (Grand Island, NY, USA). Cell culture flasks and Transwell^®^ polycarbonate inserts (12 mm diameter, 0.4 μm pore size) were obtained from Corning Costar Corp. (Bedford, MA, USA). All other chemicals were of high-performance liquid chromatography (HPLC) or analytical grade.

### 2.2. Cell Culture

Caco-2 cells between passage 40 and 50 were cultured in DMEM supplemented with 10% fetal bovine serum, 1% nonessential amino acids and antibiotics in a humidified atmosphere of 5% CO_2_ at 37 °C. The medium was changed three times a week. After reaching 85% confluence, Caco-2 cells were harvested with 0.25% trypsin-EDTA solution and seeded in Transwell^®^ inserts in 12-well plates at a density of 2.0 × 10^5^ cells/cm^2^. The culture medium was replaced every other day for the first 8 days and daily thereafter for the next 13 days until the monolayer exhibiting properties that closely resemble the morphologic and functional characteristics of normal enterocyte. The monolayer integrity of Caco-2 cells was evaluated by measuring the transepithelial electrical resistance (TEER) with a Millicell ERS volt/ohmmeter from Millipore (Bedford, MA, USA) according to the manufacturer’s instructions. Only TEER values above 300 Ω·cm^2^ at 37 °C were used for transport experiments [22].

### 2.3. Cell Viability Assay

The MTT assay was performed to evaluate the effect of Cy-3-G on cell viability, as described previously [23]. Briefly, Caco-2 cells were cultured in 96-well plates at a density of 1.0 × 10^4^ cells/well. Following the treatment of the cells with or without Cy-3-G for 24 h, MTT was added and incubated for 4 h for the formation of formazan. After the addition of DMSO to dissolve formazan crystals, absorbance of formazan was measured at 570 nm. The percentage of cell viability was calculated by comparing the absorbance of Cy-3-G treated cell with the absorbance of cells exposed to the negative control.

### 2.4. Transport Studies

To measure the transportation of Cy-3-G across the Caco-2 cell monolayer, both sides of the Transwell were washed twice and equilibrated for 30 min with pre-warmed phosphate-buffered saline (PBS). Transport studies were undertaken in the absorptive and efflux direction, separately. The transport buffer containing Cy-3-G was added to either the apical (0.5 mL) or the basolateral (1.5 mL) side of the inserts, while the receiving compartment contained the corresponding volume of transport buffer. After incubation of 30, 60, 90 and 120 min, 50-μL samples were taken from the receiver chambers and immediately replenished with an equal volume of pre-warmed PBS. The samples were acidified with an equal volume of 5% formic acid and frozen until analysis.

Inhibition studies of Cy-3-G across Caco-2 cell monolayers were investigated using an inhibitor, phloridzin or phloretin. The experiments were conducted by using a phloridzin or phloretin solution instead of blank PBS.

### 2.5. RNA Interference

To knockdown SGLT1 and GLUT2 expression, we performed transfection of human SGLT1 and GLUT2 small interfering RNA (sc-61538, sc-35495) with Caco-2 cells on Day 18 after differentiation, respectively. One-point-eight microliters of transfection reagents were added to 2.0 mL of DMEM serum-free medium containing 2 nmol/L of each siRNA oligo, incubated for 20 min and then added to the 12-well Transwell containing 1.0 mL fresh medium. A nonrelated, scrambled siRNA (sc-37007) was used as a control. Transfection reagent and all siRNA oligos were designed and synthesized by Santa Cruz. Twenty four, 48 and 72 h post-transfection, western blotting and real-time PCR were used to measure intracellular SGLT1 and GLUT2 levels.

### 2.6. Western Blotting Analysis

Cells were washed twice with ice-cold PBS and harvested in cell lysis buffer (Beyotime, Haimen, China). Protein concentrations were determined by using the bicinchoninic acid protein assay reagent kit (Thermo Scientific, Rockford, IL, USA). Equal amounts of proteins (40 μg) were separated by SDS-polyacrylamide gel electrophoresis. Western blotting experiments were performed as described previously [24]. The specific antibodies used included SGLT1, GLUT2 (Millipore, Billerica, MA, USA) and β-actin (Cell Signaling Technology, Danvers, MA, USA). We probed samples with primary antibodies and detected with horseradish peroxidase conjugated secondary antibodies using the ECL detection system (Santa Cruz Biotechnology, Santa Cruz, CA, USA). Band densities were quantified using an image analyzer Quantity One (Bio-Rad, Richmond, CA, USA). All protein quantifications were adjusted according to their corresponding β-actin level, which was not varied with different treatment conditions.

### 2.7. Quantification of SGLT1 and GLUT2 mRNA

Total RNA was isolated from Caco-2 cells using TRIzol reagent (Invitrogen, Carlsbad, CA, USA). For reverse transcription, 1.0 μg of total RNA was converted to first-strand complementary DNA in 20-μL reactions using a complementary DNA synthesis kit (Fermentas, Shenzhen, China). Quantitative real-time polymerase chain reaction (PCR) analyses were performed using SYBR Green MasterMix (Invitrogen, Carlsbad, CA, USA) in a real-time PCR machine (ABI 7500, Applied Biosystem, Foster, California, USA). Primers used in the present study are as follows: SGLT1, 5′-AAGGTTGTTTATCCTGGTGCTG-3′ (forward), 5′-TGAAATCCCAATCAGAAGTCCT-3′ (reverse); GLUT2, 5′-TGAACTGCCCACAATCTCATAC-3′ (forward), 5′-ATACAGACAGGGACCAGAGCAT-3′ (reverse). The thermal cycling program was as follows: 10 min at 95 °C for enzyme activation and 40 cycles of denaturation for 15 s at 95 °C, 1 min at 60 °C for annealing and extension, respectively. Relative transcript abundance was determined by using the ΔΔ*C*_t_ method after normalization with β-actin. The purity of PCR products was verified by melting curves and gel electrophoresis.

### 2.8. Absorption of Cy-3-G after RNA Interference

After RNA interference for 48 h, transport studies were carried out as described above. Cy-3-G (0.5 mL) was added to the apical side of the inserts, while PBS (1.5 mL) was added to the basolateral side. At different times after incubation, 30, 60, 90 and 120 min, 50-μL samples were taken from the receiver chambers, and the chamber was immediately replenished with an equal volume of pre-warmed PBS. The samples were acidified with the equal volume of 5% formic acid and frozen until analysis.

### 2.9. LCMS Analysis of Cy-3-G Content

The content of Cy-3-G was quantified by an Agilent 1200 series high-performance liquid chromatography coupled to an Agilent 6410 triple quadrupole mass spectrometer (LCMS) and an Agilent Zorbax SB-C18 column (2.1 mm × 50 mm, 1.8 μm). The solvents were A (5% formic acid in water, v/v) and Solvent B (acetonitrile); the gradient elution program was performed as follows: 2% B, 0–1 min; 2%–8% B, 2–3 min; 8%–10% B, 3–10 min; 10%–2% B, 10–15 min. The flow rate was 0.2 mL/min. The injection volume was 5 μL.

The MS detection was performed by acquiring data in the positive ion mode for Cy-3-G (449 > 287). Electrospray ionization (ESI) was performed with the following spray chamber conditions: drying gas flow of 8.0 L/min, nebulizer pressure of 40 psi and drying gas temperature of 350 °C, applying a voltage of 5000 V. Cy-3-G content was quantified by calibration curves obtained with the standard substance.

### 2.10. Statistical Analysis

All results are expressed as the means ± SD (*n* = 6). Data were statistically analyzed with either the Student’s *t*-test or one-way ANOVA. Differences were considered statistically significant if *p* < 0.05. SPSS 17.0 package (SPSS Inc., Chicago, IL, USA) was used for all statistical analyses.

## 3. Results

### 3.1. Cytotoxicity of Cy-3-G to Caco-2 Cells

In this study, we first determined the cytotoxicity of Cy-3-G by treating Caco-2 cells with various concentrations of Cy-3-G for 24 h followed by the MTT assay. Compared with the control group, cell viability was not significantly altered by Cy-3-G treatment (Supplementary Data), at concentrations range from 0 to 80 μmol/L. A noncytotoxic concentration of Cy-3-G was used in the follow-up experiments.

### 3.2. Transport of Cy-3-G in Caco-2 Cell Monolayer

The absorption of Cy-3-G was studied by using confluent and differentiated Caco-2 cell monolayers. As shown in Table 1, transport rate of Cy-3-G crossing Caco-2 cell monolayers in the apical to basolateral (A → B) direction was much higher than that in the basolateral to apical (B → A) direction at a concentration range of 10–40 μmol/L.

**Table 1:** Transport parameters of Cy-3-G across Caco-2 cell monolayer (*n* = 6).

Cy-3-G (μmol/L)	P_app_ (×10^−7^ cm/s)		Efflux Ratio	Transport Efficiency (%)
	B → A	A → B		
10	10.57 ± 1.12	14.96 ± 1.88	0.71	2.41
20	6.45 ± 0.46	8.98 ± 1.12	0.72	1.45
40	3.58 ± 0.37	4.75 ± 0.51	0.75	0.76

**Figure 1:**
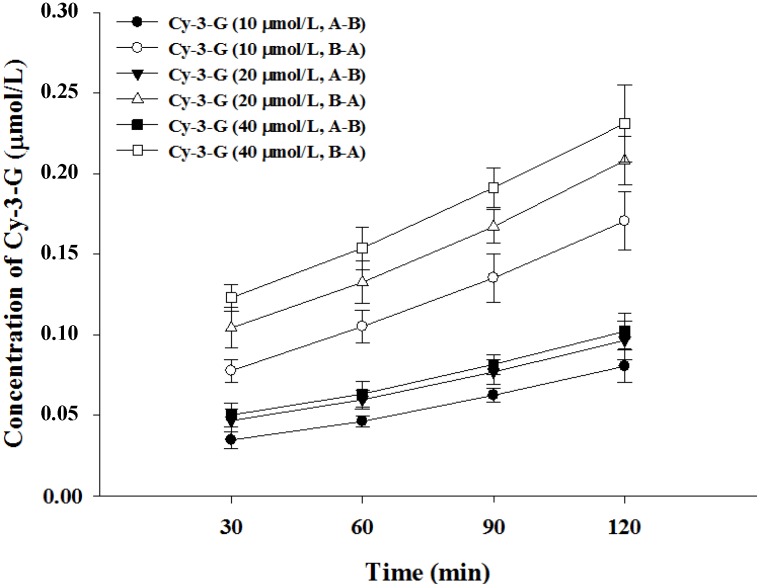
Transport of Cy-3-G in Caco-2 cells; samples were collected from 30 to 120 min.

As shown in Figure 1, the transepithelial transport in the apical to basolateral (A → B) direction was saturated when the Cy-3-G concentration was 20 μmol/L. The results suggested that the transport of Cy-3-G from apical to basolateral was mediated by transporters.

**Figure 2:**
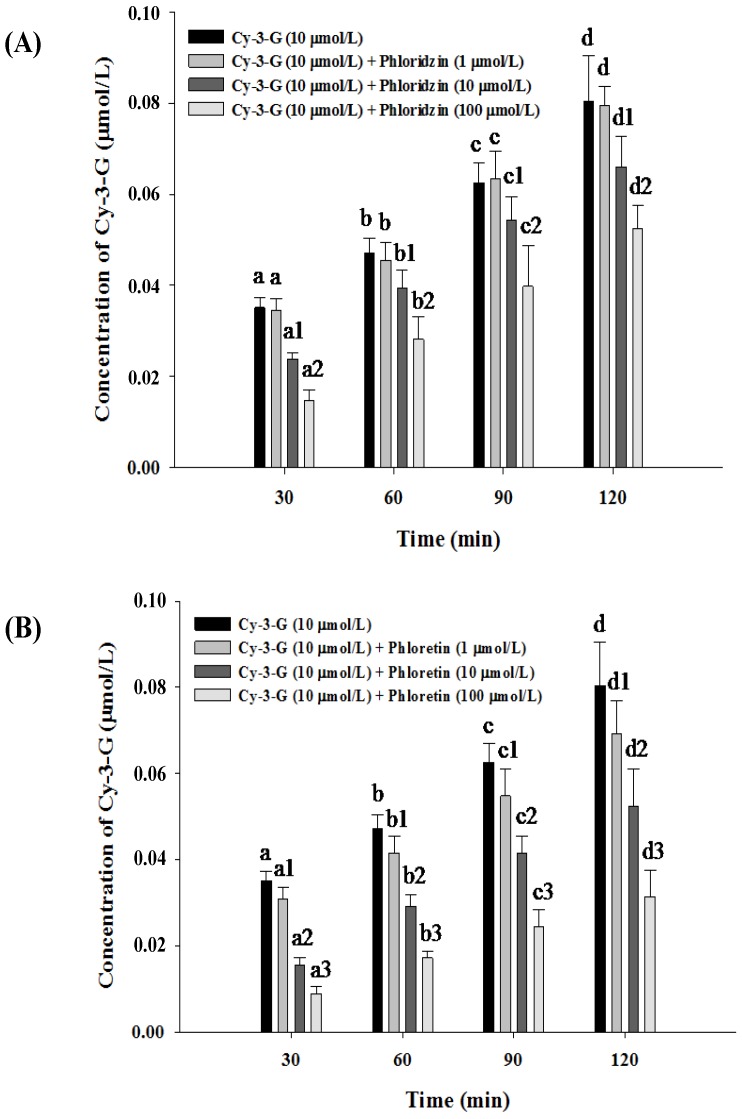
Effects of phloridzin and phloretin on Cy-3-G absorption. The cell monolayer was treated with phloridzin (**A**) or phloretin (**B**) for 0.5 h before the assays were carried out. Values without a common letter differ, *p* < 0.05.

### 3.3. Absorption of Cy-3-G in the Presence of either Phloridzin or Phloretin

The results of the effect of phloridzin on the absorption of Cy-3-G from Caco-2 cell monolayer over time are shown in Figure 2A. In the control group, Cy-3-G significantly increased in the basolateral compartment from 30 to 120 min. The addition of 1 μmol/L phloridzin did not seem to inhibit the absorption of Cy-3-G, but higher concentrations of phloridzin (10, 100 μmol/L) showed a significant effect on Cy-3-G absorption over the experimental period of 120 min.

In addition, we found phloretin to also affect the absorption of Cy-3-G (Figure 2B). In the control group, the amount of Cy-3-G increased significantly in the basolateral compartment from 30 to 120 min, compared with the control. Phloretin obviously inhibited the absorption of Cy-3-G, especially at 100 μmol/L. Increasing concentrations of phloretin showed a significant effect on Cy-3-G absorption over the experimental period of 120 min.

**Figure 3:**
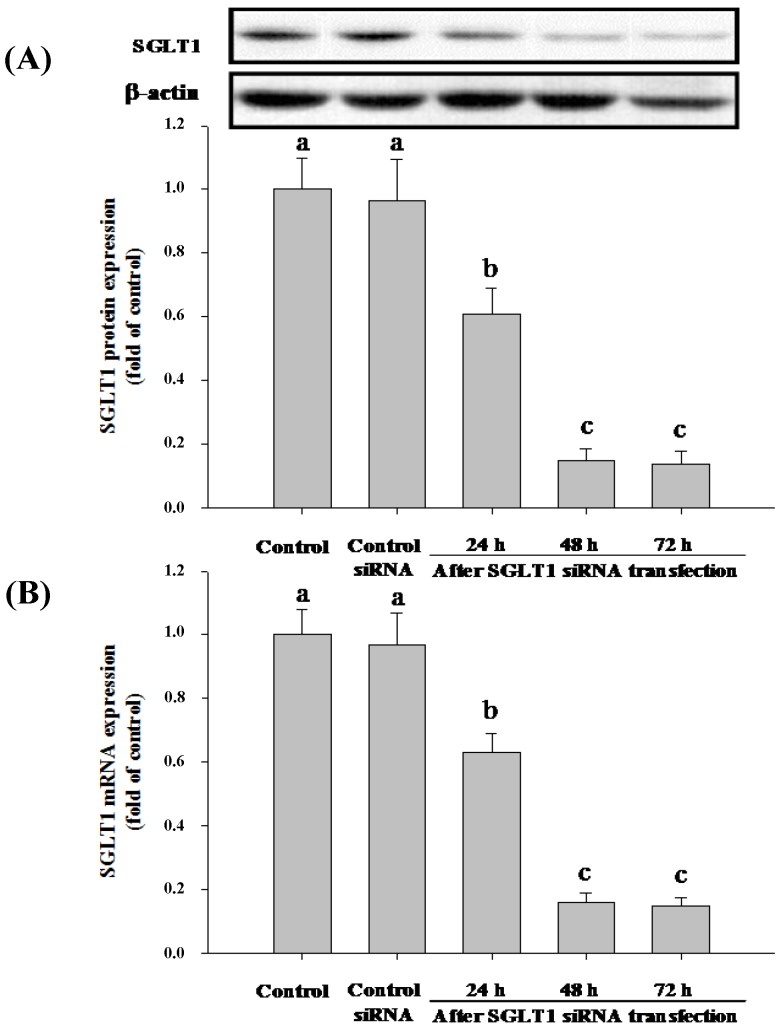
Expression of SGLT1 in Caco-2 cells after siRNA transfection. Caco-2 cells were treated with SGLT1 siRNA for 24, 48 and 72 h. Comparative expression of SGLT1 was then assessed by western blotting (**A**) and real-time PCR (**B**), respectively. Values without a common letter differ, *p* < 0.05.

### 3.4. SGLT1 and GLUT2 Involve the Absorption of Cy-3-G in Caco-2 Cells

**Figure 4:**
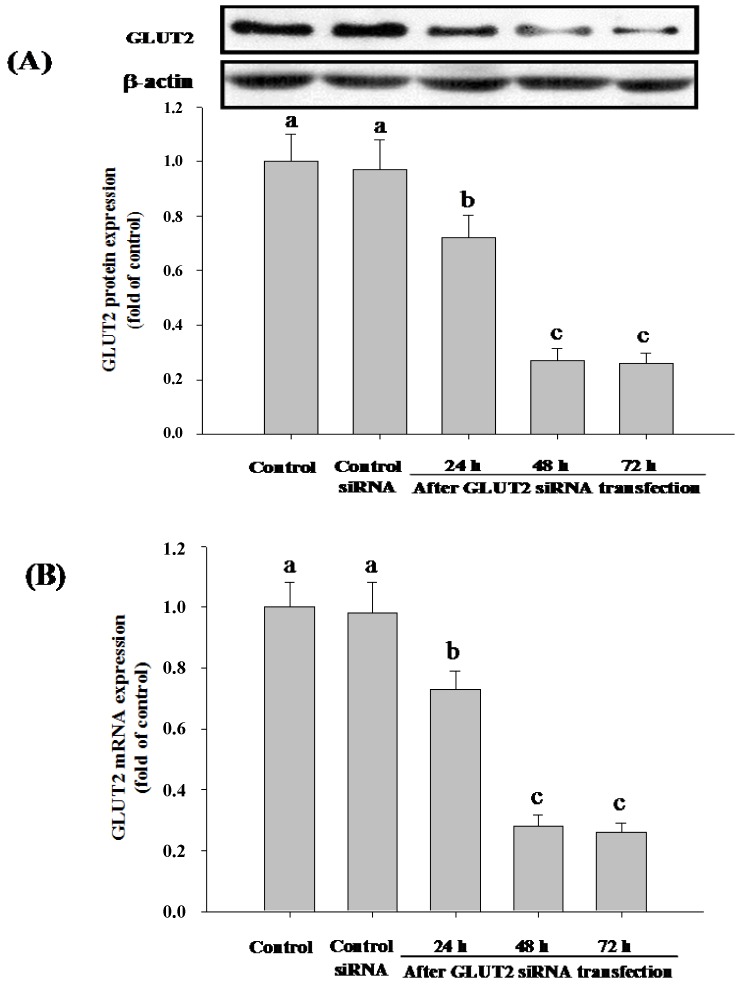
Expression of GLUT2 in Caco-2 cells after siRNA transfection. Caco-2 cells were treated with GLUT2 siRNA for 24, 48 and 72 h. Comparative expression of GLUT2 was then assessed by western blotting (**A**) and real-time PCR (**B**), respectively. Values without a common letter differ, *p* < 0.05.

Caco-2 cells express many glucose transporters, including SGLT1, GLUT1, GLUT2, GLUT3 and GLUT5. To gain better insights into the absorption mechanism of Cy-3-G, we treated Caco-2 cells with siRNA specific either to SGLT1 or GLUT2 to deplete the cells of either of these enzymes, and then, the cells were assayed for Cy-3-G absorption. The results demonstrated that after 48 h, SGLT1 siRNA exerted a remarkable decreasing of SGLT1 protein (Figure 3A) and mRNA (Figure 3B) expression; GLUT2 siRNA significantly decreased the expression of GLUT2 protein (Figure 4A) and mRNA (Figure 4B).

**Figure 5:**
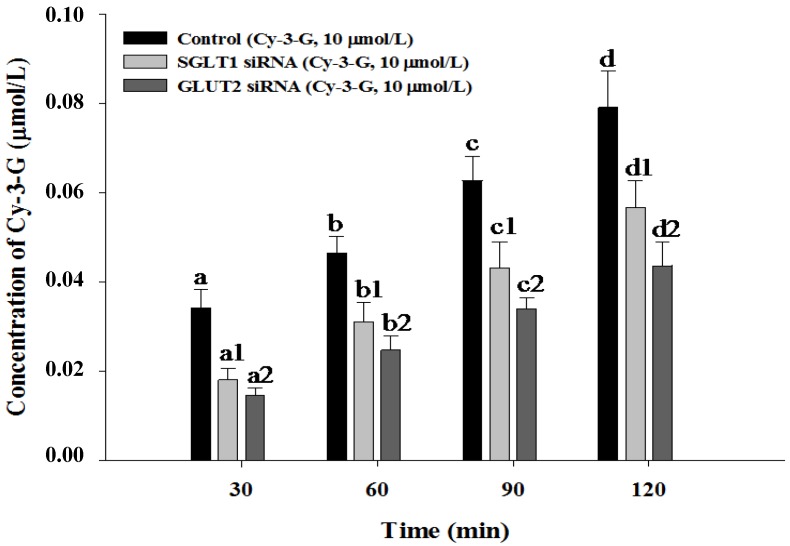
Absorption of Cy-3-G in Caco-2 cells after siRNA transfection. After transfection for 48 h, Cy-3-G absorption was measured. Values without a common letter differ, *p* < 0.05.

Then, we investigated Cy-3-G absorption across Caco-2 cells. As shown in Figure 5, Cy-3-G absorption across Caco-2 cells gradually increased from 30 to 120 min in the control group. However, after SGLT1 or GLUT2 siRNA transfection in Caco-2 cells, the transport rate of Cy-3-G was significantly decreased from 30 to 120 min compared with the control group (*p* < 0.05). There results show that SGLT1 and GLUT2 are involved in the absorption of Cy-3-G in Caco-2 cells.

## 4. Discussion

It is generally known that anthocyanins are beneficial to human health. We therefore considered it important to increase our knowledge on the mechanisms of their absorption. The long-term view that anthocyanins could only be absorbed in their aglycon forms [14,25] has been modified in that anthocyanins are unique compared with other flavonoids, since that they are absorbed in intact glycosylated forms. The intact anthocyanins were detected in the plasma and urine of human subjects and rats [16,17,26]. In our previous study, apoE-deficient mice were orally gavaged with Cy-3-G; the plasma concentration of Cy-3-G reached the maximum at 0.5 h and was undetectable at 6 h. This is consistent with others studies, but its absorption mechanism in the small intestine is unclear.

Compared to human and animal models, which are highly complex and can be easily influenced by other factors, an *in vitro* model is much easier to control. It has been reported that Caco-2 cells can undergo spontaneous differentiation under certain culture conditions and exhibit characteristics of mature enterocytes [27]. The cell surface that faces the top medium develops a brush border that resembles the luminal membrane of the intestinal epithelium. The cell surface that attaches to the permeable membrane and faces the bottom medium develops into the basolateral membrane. It is a well-established model to study intestinal absorption [28].

In the present study, Caco-2 cells between passage 40 and 50 were selected, because their biochemical, morphological and transport characteristics are relatively stable within this range. The results showed that Cy-3-G could be transported through the Caco-2 cell monolayers in intact glycone forms, although the absorption efficiency was relatively low. The hypothesis raised was that anthocyanins could interfere with the transporters responsible for their own transport. Candidates for anthocyanin transporters were the glucose transporters, since anthocyanins possess a sugar moiety, in particular a glucose residue. It has been suggested that glucoside flavonoids, such as quercetin-3-glucose, could enter the cell anchored by the glucose residue, using hexose transporters [29].

SGLT1 and GLUT2 are the main hexose transporters described in Caco-2 cells. SGLT1 is an energy-dependent and sodium-dependent cotransporter and present only on the apical membrane. GLUT2 is a facilitated transporter. Until a few years ago, GLUT2 was described to be present only in the basolateral membrane and in some pathologies on the apical membrane [30]. Recently, it has been described and accepted that GLUT2 is present on the apical side and can be recruited to the membrane in the presence of a great amount of glucose, thus becoming the main transporter responsible for glucose uptake [31]. Some studies reported that monoglucosides of flavonoids can be transported across the apical membrane of enterocytes by the sodium-dependent glucose transporter, SGLT1 [29,32]. Mailleau *et al.* [33] reported that SGLT1 activity rapidly increased from Day 12 up to Day 20 after seeding of Caco-2 cells. Milbury *et al.* [34] suggested that the absorption of anthocyanins in their unchanged glycosylated forms may indicate the involvement of glucose transport receptors. Mulleder *et al.* [35] observed that urinary excretion of anthocyanins in humans was reduced when sucrose was ingested together with an elderberry concentrate, indicating that anthocyanins are probably associated with SGLT1. Walton *et al.* [36] found that quercetin-3-glucoside inhibits Cy-3-G absorption *in vitro*, probably by competing for the same transporter. This would be in agreement with our findings that Cy-3-G absorption was inhibited by varying concentrations of phloridzin or phloretin at each time point. Moreover, we found that Cy-3-G absorption decreased after SGLT1 or GLUT2 siRNA transfection. Hence, SGLT1 and GLUT2 are probably involved in the intestinal absorption of Cy-3-G.

## 5. Conclusions

Our data demonstrate that SGLT1 and GLUT2 play a role in the absorption of Cy-3-G in a Caco-2 cell monolayer. The results from studies with pharmacological inhibitors have also been supported by studies using siRNA to make Caco-2 cells specifically deficient in either SGLT1 or GLUT2. The present findings suggest that SGLT1 and GLUT2 could be a limiting step for the bioavailability of Cy-3-G.

## Supplementary Files

Supplementary File 1
