# Supplementary material for: The Role of Sodium-Dependent Glucose Transporter 1 and Glucose Transporter 2 in the Absorption of Cyanidin-3-O-β-Glucoside in Caco-2 Cells
Source: Nutrients. 2014 Oct 13;6(10):4165–77. doi: 10.3390/nu6104165 (PMC4210911; doi:10.3390/nu6104165)
Supplement: Supplementary File 1 [file nutrients-06-04165-s001.docx]

**Supplementary Information**

**Supplementary Data.** Effect of Cy-3-G on the viability of Caco-2 cells. Cells were treated with 0–80 µmol/L Cy-3-G for 24 h before being subjected to a MTT assay.
